# Kinetic parameters of *Scenedesmus obliquus* (Chlorophyceae) growth and yield under different cultivation conditions *in vitro*

**DOI:** 10.7717/peerj.17879

**Published:** 2025-08-11

**Authors:** Arialdo M. Silveira Júnior, Silvia Maria Mathes Faustino, Fabrício Holanda e Holanda, Irlon Maciel Ferreira, Alan Cavalcanti da Cunha

**Affiliations:** 1 Department of Environment and Development, Federal University of Amapá, Macapá, Amapá, Brazil; 2 Postgraduate Program in Tropical Biodiversity, Federal University of Amapá, Macapá, Amapá, Brazil; 3 Department of Biological and Health Sciences, Federal University of Amapá, Macapá, Amapá, Brasil; 4 Biocatalysis and Applied Organic Synthesis Group, Federal University of Amapá, Macapá, Amapá, Brazil; 5 Department of Exact and Natural Sciences, Federal University of Amapá, Macapá, Amapá, Brazil

**Keywords:** Microalgae, NPK, Growth kinetics

## Abstract

The aim of the present study is to assess the kinetic growth and yield of microalgae belonging to the species *Scenedesmus obliquus* (Kützing, 1833) grown under different macronutrient concentrations (N:P:K), pH and temperature. A three-level three-variable Box-Behnken factorial design was developed to test the influence of environmental factors on the growth parameters applied to a microalgal crop, namely: maximum cell density (N_max_), specific growth rate (µ, d^−1^), doubling time (T_2_) and maximum yield (P_max_). A classical logistic growth model was applied to estimate the kinetic behavior of a *S. obliquus* culture in comparison to other studies in the literature. Maximum cell density (N_max_) significantly changed between 32.3 ± 0.84 and 101.5 ± 12.5 × 105 cells mL^−1^ (*p* = 0.004). Specific growth rates (µ) and P_max_ showed significant mean variations between 0.28 ± 0.10 day^−1^ and 6.21 ± 4.57 cells mL^−1^ d^−1^, respectively. All growth curves were adjusted to the proposed logistic model and range variation was (0.65 ≤ r^2^ ≤ 0.99, *p* = 0.01). Maximum microalgae cell density per crop was found at temperature of 24 °C (N_max_ = 49.0 × 105 cells mL^−1^) (*p* = 0.01), in 20 mL Nitrogen, Phosphorus, and Potassium (NPK) dilution (N_max_ = 46.5 × 105 cells mL^−1^) (*p* = 0.02). Despite the significant variation in pH values (5.0 ≤ pH ≤ 9.0, *p* = 0.05), it did not have strong influence on plant growth responses. In conclusion, the kinetic study applied to *S. obliquus* growth and yield parameters emerged as reference for this species’ kinetic behavior and bioprospecting under controlled and experimentally standardized conditions, and as support for pilot projects on bioreactors.

## Introduction

Microalgae are a source of organic compounds that present high potential for bioprospecting and biotechnological applications, mainly for producing both biodiesel and dietary supplements used in human and animal nutrition (Breuer et al., 2012; Moreno-Garcia et al., 2017). Silveira Júnior, Faustino & Cunha (2019) conducted a literature review to analyze the role played by microalgae in bioprospecting and their application as dietary supplement and immunostimulant at regional and global level, with emphasis on aquaculture in the Brazilian Amazon. They assessed the primary advantages of applying microalgae bioactive compounds and found gaps in the knowledge about this topic. These gaps assumingly hinder the economic and biotechnological exploitation of these microalgae.

The increased production of microalgal biomass grown in cultivation ponds (open systems) and in photobioreactors (closed systems) has been a trend for some decades (Posten, 2009; Silveira Júnior, Faustino & Cunha, 2019; Wen et al., 2016). These crops have enhanced the use of these microalgae in the food, aquaculture, and bioenergy industries (Adams et al., 2013; Hempel, Petrick & Behrendt, 2012; Mallick et al., 2016; Wen et al., 2016). However, despite such a production trend, knowledge on its high operational costs and on these organisms’ industrial use, mainly on their yield and effective biomass production, remains scarce (Tan & Lee, 2016; Wang, Sheng & Yang, 2017). Conditioning factors associated with these processes need to be better understood to allow foreseeing the specific parameters to make its sustainable production feasible, mainly when it comes to its multiple biotechnological applications (Barbera et al., 2016).

Microalgal production stages must be optimized in order to achieve appropriate harvesting, drying and biomass extraction conditions (Valverde et al., 2016). However, yield optimization and associated cost reductions remain a challenge due to difficulties in controlling basic conditions and microalgal growth specifications (Lee, Jalalizadeh & Zhang, 2015; Silveira Júnior, Faustino & Cunha, 2019) that, in their turn, limit and hinder the development of new research. Cultivation method optimization has evolved over the last few years (Barbera et al., 2016; Bohnenberger & Crosseti, 2014; Hodaifa et al., 2010; Sipaúba-Tavares et al., 2017; Xu et al., 2012), but kinetic studies carried out with microalgae must be encouraged (Silveira Júnior, Faustino & Cunha, 2019). These authors acknowledge the urgency in building a biotechnological basis, such as microalgal growth kinetic analysis, to support the development of photobioreactor projects. In addition, kinetic parameters are the starting point for process design and control, since they allow better operational cultivation performance and yield (Lee, Jalalizadeh & Zhang, 2015; Surendhiran et al., 2015).

Some classical studies on microalgae kinetic parameters are associated with both single and multiple factors that, in their turn, influence microalgal growth. Light intensity, pH, nutrient availability, dissolved CO_2_ and O_2_ concentration, and temperature are essential parameters for microalgae crops (Hodaifa et al., 2010; Krzemińska et al., 2014; Lee, Jalalizadeh & Zhang, 2015; Li, Gao & Lin, 2015). Assessing these parameters’ influence on microalgae growth kinetics is a basic condition to reach a successful cultivation, since they define the optimal or most appropriate conditions for different cultivation alternatives. Understanding constraints imposed by these key parameters is the way to accomplish better biomass growth and yield rates (Arumugam et al., 2013; Baumgartner et al., 2013; Tan & Lee, 2016).

*S. obliquus* has been extensively investigated in bioprospective studies. Zaharieva et al. (2022) assessed the effect of luminosity on specific growth rates and biocompound production in *S. obliquus* biomass. They observed higher content of polyphenols under red light, whereas the green light favored flavonoids accumulation in the biomass. These compounds enhanced antimicrobial effects against foodborne pathogens when they were associated with penicillin, fluoroquinolones or with oregano essential oil, at concentrations ranging from 0.01 to 0.05 mg/mL. Kaewkannetra, Enmak & Chiu (2012) assessed salinity effect on *S. obliquus* cultures in the presence of NaCl, at concentrations of 0.05, 0.2, and 0.3 M. The highest lipid accumulation was found in cells grown under saline stress in comparison to that recorded in non-saline medium, and it led to 36% lipid content. According to Gorrini et al. (2020), *S. obliquus* biomass production can be significantly enhanced by properly manipulating control variables, such as incident light, biomass and substrate concentrations. These parameters work as natural and effective control strategy for optimization issues linked to this species’ culture.

The main hypothesis tested in the present study lied on finding the basic limits for parameters applied to control the kinetic growth behavior of *S. obliquus* grown in photobioreactors, at bench scale. The tested kinetic model had to keep first-order reaction features and they can be defined through a classic logistic model, at relatively high reliability (r^2^ > 75%). Experimental adjustments to data of the proposed model pointed out that the kinetic growth behavior of the assessed microalgal species can be compared to behaviors observed in similar studies available in the literature (Arumugam et al., 2013; Barbera et al., 2016; Goswami & Kalita, 2011; Nayak, Thirunavoukkarasu & Mohanty, 2016; Sipaúba-Tavares et al., 2017; Xin et al., 2010). Therefore, it was safe and replicable at pilot scale level, if one takes into consideration similar and controlled environments.

The aim of the current investigation was to test a classical logistic growth model to represent *S. obliquus* growth kinetics at adequate significance level. This microalgae species is naturally distributed in Amazonian ecosystems. The main aims of the present study were to test growth and yield rate variations, and to assess different cultivation conditions (Nitrogen, Phosphorus, and Potassium (NPK) concentration and dilution, pH and temperature) as limiting or potentiating factors influencing kinetic microalgal growth in crop systems, *in vitro*, to assess its sustainable production at industrial scale.

## Materials & Methods

### *Scenedesmus obliquus* isolation and culture activation

*Scenedesmus obliquus* isolation was performed based on both capillarity and successive dilution techniques (Lourenço, 2006). The experiment was carried out at the Algae Cultivation and Bioprospecting Laboratory (LACAL) of Federal University of Amapá (UNIFAP), after the species was subjected to taxonomic identification through optical microscopy. Phytoplankton samples collected in a natural aquatic ecosystem (Lagoa dos Índios, Amapá, Brazil, 0°01′54.260″N 51°06′09.665″W) were used in the study. Isolated cells were kept in test tubes filled with liquid synthetic culture medium (NPK 15:05:05) (Table 1). Cells were incubated at 23 °C, under continuous photoperiod. Isolates (11.7 ± 0.3 × 10^4^ cells mL^−1^) were transferred to activation crops in Erlenmeyer (200 mL) flasks until the tests were run.

**Table 1:** Culture medium used for *Scenedesmus obliquus* microalgae culture.

		**NPK dilution (mg/L)**		
**Medium**	**Reagent**	**12**	**16**	**20**
NPK	Nitrogen (water-soluble N)	0.64 g/L	0.86 g/L	1.12 g/L
	Phosphorus (water-soluble P_2_O_5_)	0.19 g/L	0.25 g/L	0.32 g/L
	Potassium (water-soluble K_2_O)	0.26 g/L	0.35 g/L	0.44 g/L

### Experimental design and *S. obliquus* cultivation in photobioreactor

A three-level three-variable Box-Behnken factorial design was developed to determine the temperature (21 °C, 24 °C and 28 °C), pH (5.0, 7.0 and 9.0) and NPK dilution (12 mg/L, 16 mg/L and 20 mg/L) influence on *S. obliquus* cultivation (Table 2). The pH range was defined according to conditions naturally observed in Amazonian ecosystems (Barros et al., 2021). STATISTICA^^®^^ software (version 10, Statesoft–Inc., Tulsa, USA, trial version, 2011) was used in the experimental design for matrix elaboration. Independent variables were divided into X1, X2 and X3.

**Table 2:** Variables used in Box-Behnken factorial experimental planning.

**Factor**	**Variable**	**Level**		
		**−1**	**0**	**+1**
X*1*	pH	5	7	9
X*2*	Temperature (°C)	21	24	28
X*3*	NPK dilution (mg/L)	12	16	20

Inoculates (200 mL) were transferred to bioreactors—2L Erlenmeyer vials (*in vitro*) filled with NPK culture medium at different dilutions (12, 16 and 20 mg/L distilled water)—at the beginning of the experiments (Table 2). Initial axenic inoculum consisted of 11.7 ± 0.3 × 10^4^ cells mL^−1^ in all assays. All experiments were simultaneously conducted in triplicate.

Inoculates were grown and kept under continuous photoperiod (28.5 µmol m^−2^s^−1^), as already reported for *S. obliquus* (Breuer et al., 2013; Girard et al., 2014). Constant aeration was carried out by CO_2_ (14 L min^−1^) air diffusion through pumping (1/2 HP). Temperature in the growing room was controlled and monitored with the aid of digital thermometers (21 ± 0.4 °C, 24 ± 0.6 °C, 28 ± °0.9 °C). Different pH values were determined by adding HCl 1N and NaOH 1N (5 ± 0.7, 7 ± 0.5 and 9 ± 0.3) solutions. The pH in the culture medium was measured every 48 h with the aid of a pH-meter.

### Cell density and yield

Cell density in the culture (average recorded for the three replicates) was measured on a daily basis in Neubauer hemocytometer by counting microalgal cells in optical microscope (Lourenço, 2006). Density values were used to calculate specific growth rate (µ), maximum cell concentration (N_max_) and maximum yield (P_max_).

Specific growth rate (µ, d^−1^) was calculated based on the logarithmic phase regression of the cell growth curve. Maximum cell concentration (N_max_, cells mL^−1^) was represented by the maximum concentration value recorded in the logarithmic phase. Maximum yield (P_max_, cel mL^−1^ d^−1^) was calculated through Eq. (1):

(1) \begin{eqnarray*}{p}_{max}= \frac{({X}_{1}-{X}_{0})}{({t}_{1}-{t}_{0})} \end{eqnarray*}

wherein, X_1_ is cellular concentration (cells mL^−1^) at time t_1_ (d) and X_0_ (cells mL^−1^) is cellular concentration at time t_0_ (d) (Schmidell et al., 2001). Doubling time per day (T_2_) was calculated through Eq. (2):

(2) \begin{eqnarray*}{T}_{2}= \frac{ln2}{\mathrm{\mu }} .\end{eqnarray*}

### Cellular density logistic model adjustment

Microalgae growth kinetics can often be modeled through the Verhulst logistic equation (Mazumdar et al., 2018; Yang et al., 2011), which is a model based on three important parameters: initial cell density at T_0_ (cells mL^−1^), theoretically reached maximum cell density (cells mL^−1^) and specific growth rate (µ, day^−1^).

Cell density (N_t_), at any time (t), based on N_o_ as the initial cell density is given by Eq. (3) in this model:

(3) \begin{eqnarray*}{N}_{t}= \frac{{N}_{0}.{N}_{Max}}{{N}_{0}+({N}_{Max}-{N}_{0}){e}^{-\mathrm{\mu }\mathrm{t}}} .\end{eqnarray*}

A transformed version of this Eq. (3) was used to estimate the growth parameters. It was based on adjusting the experimental raw data in the model of a nonlinear function (logistics) through the least square’s method (Mazumdar et al., 2018; Weisberg, 2005), which is expressed in Eq. (4):

(4) \begin{eqnarray*}{N}_{t}= \frac{{N}_{Max}}{1+{e}^{(\mathrm{\theta }+\mathrm{\mu }\mathrm{t})}} \end{eqnarray*}

wherein, N_t_ is cell count at time t, t is time after experiment has started, N_Max_ isthe maximum estimated cell count, N_0_ is the initial cell count, µis maximum specific growth rate - *θ* was defined through Eq. (5):

(5) \begin{eqnarray*}\theta =\mathrm{ln}( \frac{{N}_{0}}{{N}_{Max}-{N}_{0}} ).\end{eqnarray*}

### Statistical treatment

Model significance was tested in the R Statistic (version R Core Team, 2016) software. Logistic regression analysis was applied to assess the explainability and significance of models adjusted based on Eqs. (2) and (3).

Shapiro–Wilk normality test, followed by Levene/Bartlett tests, was carried out to assess experimental data homogeneity and homoscedasticity and to model the assessed kinetic parameters. Analysis of variance applied to repeated measurements was performed through Kruskal–Wallis test, followed by *post-hoc* dunn test, to find significant differences in growth parameters, in different experiments, since data distribution rejected normality. All tests were considered significant at *α* < 0.05.

STATISTICA software^®^ (*version 10, Statesoft - Inc., Tulsa, USA, test version, 2011*) was used for data analysis. Analysis of variance (ANOVA) was applied to transformed data to assess influence significance and interactions between independent variables, namely: temperature, pH and NPK dilution. Pareto graphs were plotted to find the significance of tested variables and to accomplish modeling through response surfaces.

## Results

### Cell density and microalgal yield

The effect of different culture medium dilution, temperature and pH levels on *S. obliquus* cultivation was tested based on logistic models defined through Eqs. (2) and (3). Significant differences were observed in all assessed microalgal growth measurements between experiments. Maximum cell density (N_max_) ranged from 32.6 to 101.5 × 10^5^ cells mL^−1^; median was 42.1 × 10^5^ cells mL^−1^. This variation led to significantly higher N_max_ in experiments 9 and 6, which were respectively defined as E9pH_9_T_24_C_20_ (101.5 × 10^5^ cells mL^−1^) and E6pH_5_T_21_C_20_ (56.2 × 10^5^ cells mL^−1^) (*p* = 0.04 and *p* = 0.004) (Table 3). Similarly, specific growth rates (µ) and maximum yield (Pmax) recorded medians of 0.24 d^−1^ and 5.46 cells mL^−1^ d^−1^, respectively. Significant and higher values were also observed in experiments 9 and 6, respectively: E9pH_9_T_24_C_20_ and E6pH_5_T_21_C_20_ (*p* = 0.002 and *p* = 0.005).

**Table 3:** Maximum cell density (N_max_), daily growth rate (µ), doubling time (T_2_) and maximum yield (P_max_) recorded for *Scenedesmus obliquus* under different growing conditions. Different letters in the same column indicate significant difference at *p* < 0.05 (Dunn’s Test).

**Order**	**Experiment**	**N**_**0**_ **(cel mL**^−1^ × **10**^**4**^**)**	**N**_**max**_ **(cel mL**^**1**^ × **10**^**5**^**)**	µ **(day**^−1^**)**	**T**_**2**_ **(day)**	**P**_**max**_ **(cel mL**^−1^ × **10**^**5**^ **d**^−1^**)**
1	E1pH_7_T_21_C_12_	11.7	48.5^abc^	0.30^a^	2.28^ac^	5.46^ac^
2	E2pH_7_T_24_C_16_	11.7	44.0^ab^	0.29^a^	2.36^a^	6.64^ac^
3	E3pH_5_T_24_C_12_	11.7	39.7^ab^	0.22^a^	3.07^ab^	3.85^ab^
4	E4pH_7_T_28_C_12_	11.7	42.1^ab^	0.24^a^	2.81^a^	4.37^ab^
5	E5pH_9_T_21_C_16_	11.7	38.8^ab^	0.13^b^	5.27^b^	5.75^a^
6	E6pH_5_T_21_C_20_	11.7	56.2^cd^	0.36^ac^	1.92^ac^	10.7^cd^
7	E7pH_9_T_28_C_16_	11.7	34.1^b^	0.21^a^	3.27^a^	2.64^b^
8	E8pH_5_T_28_C_20_	11.7	32.6^a^	0.18^a^	3.84^b^	2.83^b^
9	E9pH_9_T_24_C_20_	11.7	101.5^d^	0.48^c^	1.44^c^	17.9^d^
	Median	11.7	42.1	0.24	2.81	5.46
	*p*-value	–	*p* < 0.05	*p* < 0.05	*p* < 0.05	*p* < 0.05

Different experiments presented median cell-doubling time of 2.81 days. The longest doubling time (T_2_) was recorded for experiment 5, *i.e.,* E5pH_9_T_21_C_16_ (5.2 days) (*p* = 0.005) - values were similar to those observed for assays 3 and 8, respectively: E3pH_5_T_24_C_12_ (3.07 days) and E8pH_5_T_28_C_20_ (3.8 days) (Table 3). The shortest cell doubling time was observed for experiment 9, *i.e.,* E9pH_9_T_24_C_20_ (1.4 ± 0.17 days) (*p* = 0.002) - values were similar to those recorded for experiments 1 and 6, respectively: E1pH_7_T_21_C_12_ (2.0 ± 0.14 days) and E6pH_5_T_21_C_20_ (1.6 ± 0.19 days).

### Adjustment to the logistic model applied to microalgae cell density

**Figure 1:**
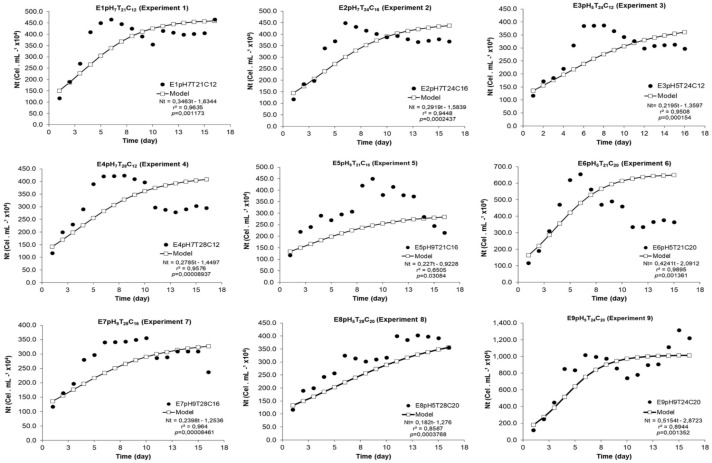
Experimental and adjusted growth curve of *Scenedesmus obliquus* grown under different cultivation conditions.

**Figure 2:**
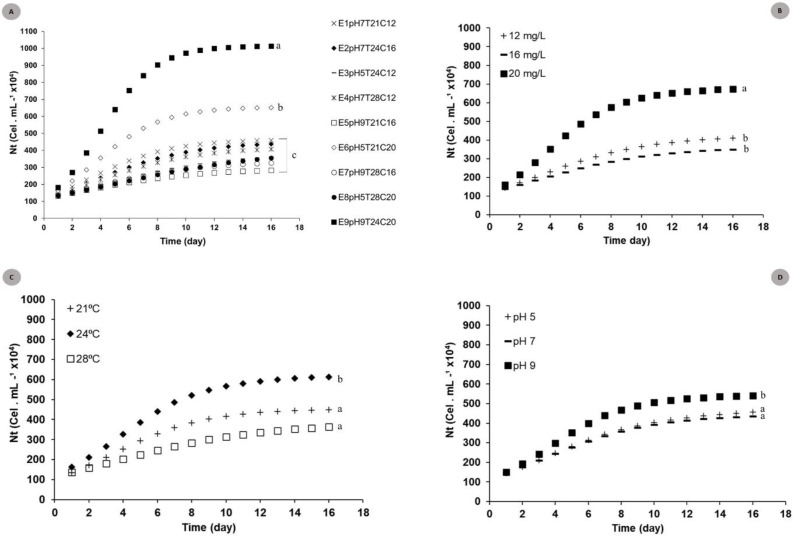
Theoretical logistic growth curves estimated for *S. obliquus* cultivated under different conditions. (A) General: curves adjusted to all experiments together; (B) Exclusive concentration influence: curves adjusted under the influence of different medium dilutions; (C) Exclusive temperature influence: curves adjusted based on the influence of different temperatures; (C) Exclusive pH influence: curves adjusted based on the influence of different pH values. Different lower-case letters indicate significant differences at *p* < 0.05.

The estimated model significantly adjusted itself to the experimental data, since r^2^ ranged from 0.65 (experiment 5) to 0.99 (experiment 6) (Fig. 1). All modeled growth curves presented noticeably short or, practically, no *lag* phase (growth induction or latency). All experiments presented exponential growth phase between the 2nd and 7th experiment days (Figs. 1 and 2). The end of the stationary phase and the start of the decline phase (cell death) in the crop were observed between the 10th and 16th day of microalgal culture. The maximum concentration phase was detected when N_Max_ was also observed.

The logistic curve adjustment showed significant differences between Nt responses recorded for the nine models tested under different pH, temperature and N:P:K concentration conditions. Experiments E9pH_9_T_24_C_20_ (N_max_ = 101.5 × 10^5^ cel mL^−1^) and E6pH_5_T_21_C_20_ (N_max_ = 56.2 × 10^5^ cel.mL^−1^) (*p* = 0.002 and *p* = 0.007) recorded the highest cell density growth expression. (Fig. 2A).

The adjusted curves showed significant differences when the different factors (temperature × pH × medium dilution) (Figs. 2B, 2C and 2D) used for factorial planning worked in the same microalgal crop growth parameters. Higher microalgaceate cell density was observed in microalgal growth at 24 °C (N_max_ = 49.0 × 10^5^ cells mL^−1^) (*p* = 0.01), in 20 mg/L culture-medium dilution (N_max_ = 46.5 × 10^5^ cells mL^−1^) (*p* = 0.02). Crops stored at pH 9 tended to present higher cell density (*p* = 0.05).

### Assessing *S. obliquus* cultivation based on response surface modeling

**Figure 3:**
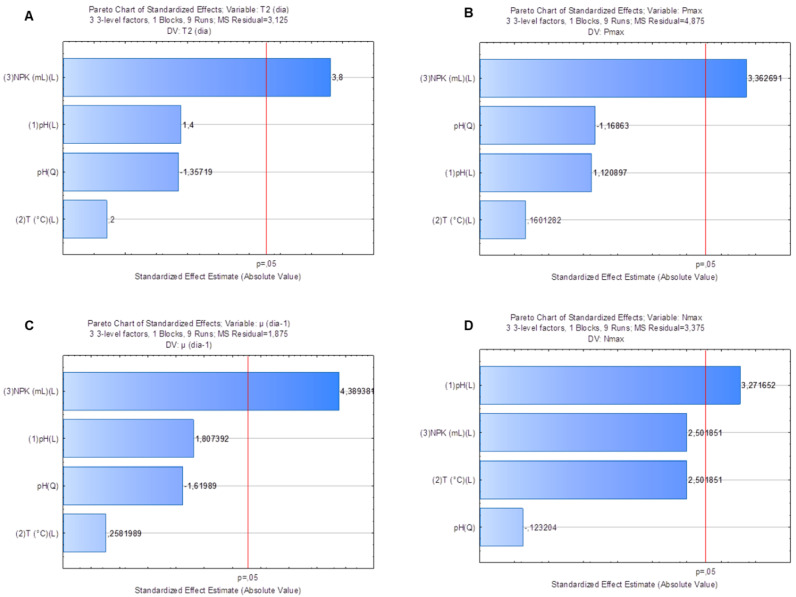
Pareto graph to find the significance (*p* < 0.05) of T_2_(3A), N_max_ (3B), Pmax (3C) and µ(day^−1^) (3D) as variable response to *Scenedesmus obliquus* cultivation under different pH, medium dilution and temperature conditions.

Factorial planning allowed assessing the influence of different factors on response variables: doubling time (T_2_), maximum cell density (N_max_), maximum yield (P_max_) and specific growth rate (µ, day^−1^). Pareto graphs pointed towards the strong effect of NPK medium dilution on variables T_2_ (Fig. 3A) (*p* = 0.02), P_max_ (Fig. 3B) (*p* = 0.03) and µ(day^−1^) (Fig. 3C) (*p* = 0.01). Analysis of variance (ANOVA) applied to these variables’ model showed coefficients of determination (r^2^) equal to 0.83, 0.79 and 0.87, respectively. Similarly, there was strong crop pH (*p* = 0.03) effect on N_max_ response in the *S. obliquus* culture (Fig. 3D). ANOVA applied to the model of variable N_max_ presented coefficient of determination (r^2^) equal to 0.87.

**Figure 4:**
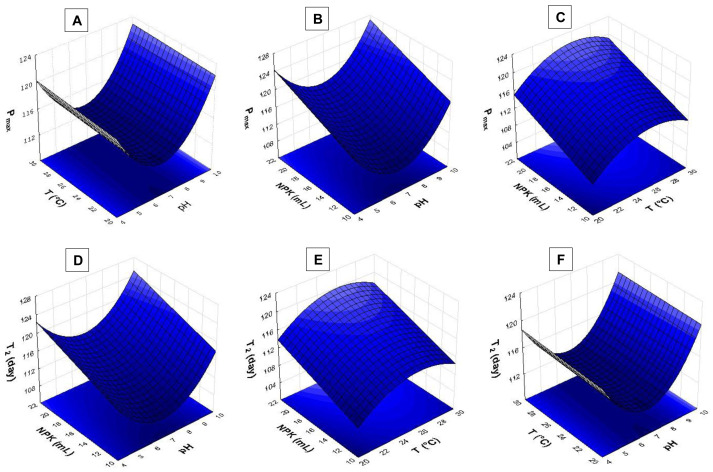
Response surface recorded for *S. obliquus* culture (A) P_max_ based on temperature and pH; (B) P_max_ based on pH value and NPK dilution; (C) P_max_ based on temperature and NPK dilution; (D) T_2_ based on pH value and NPK dilution; (E) T_2_ based on temperature and NPK dilution; (F) T_2_ based on temperature and pH.

**Figure 5:**
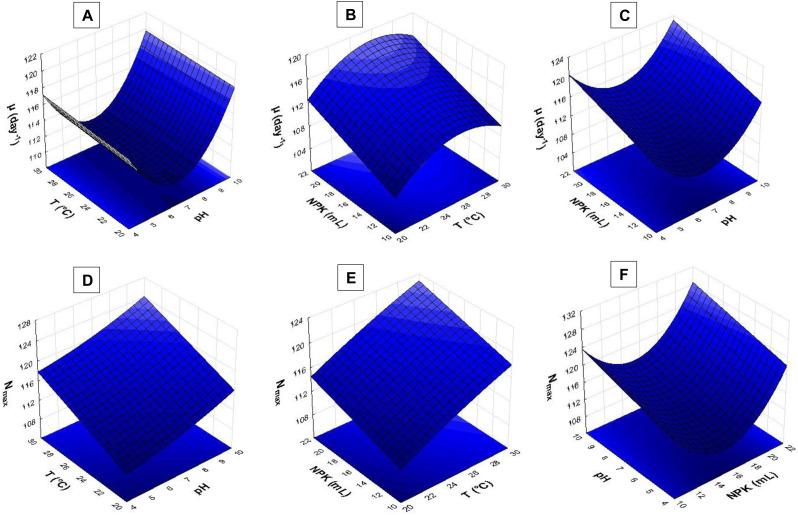
Response surface recorded for *S. obliquus* culture. (A) µ(day^−1^) based on temperature and pH; (B) µ(day^−1^) based on temperature and NPK dilution; (C) µ(day^−1^) based on pH and NPK dilution; (D) N_max_ based on temperature and pH; (E) N_max_ based on temperature and NPK dilution; (F) N_max_ based on pH and NPK dilution.

The response surface analysis (Figs. 4 and 5) showed that pH ranging from 8.5 to 9.0 had strong influence on biomass yield based on variable P_max_. Wide temperature range (21 to 28 °C) affected *S. obliquus* yield (Fig. 4A). The higher the NPK concentration in the crop, the higher the biomass yield in comparison to the effect of different pH values (Figs. 4B and 4C).

According to the response surfaces, the highest NPK concentrations had more influence on cell doubling time T_2_ than crop pH (Fig. 4D). Temperature ranging from 24 °C to 29 °C had strong effect on *S. obliquus* cell doubling when it was conditioned in 24 mL/L NPK (Fig. 4E). Temperature highly interacted with T_2_ when temperature ranged from 24 °C to 28 °C. The pH value had influence on T_2_ when it was higher than 8.5 (Fig. 4F).

The pH > 9.0 in association with temperatures higher than 24 °C had strong influence on *S. obliquus* specific growth rate (µ, day^−1^) (Fig. 5A). Growth rate was assessed based on NPK dilution and temperature (Fig. 5B), as well as on NPK and pH dilution (Fig. 5C). Higher NPK concentrations had strong influence on the specific growth rate in both response surfaces. Temperatures between 24 °C and 29 °C, in association with pH higher than 9, affected *S. obliquus* growth rate (Figs. 5B and 5C).

Response surface applied to maximum cell density (N_max_) based on crop temperature and pH showed interaction between temperatures > 27 °C and pH > 9.0 (Fig. 5D). Higher N_max_ was observed when temperature ranging from 27 °C to 29 °C was associated with higher NPK concentrations in *S. obliquus* culture (Fig. 5E). Crops conditioned at pH ranging from 7.5 to 9.5 presented high cell density when they were conditioned under the highest NPK concentration (Fig. 5F).

## Discussion

Microalgae biomass production, mainly for bioprospecting and for identifying compounds with potential to be used for several purposes, has become an attractive factor in recent decades. The growth of these microalgae is associated with their likely large-scale cultivation, based on their biomass extraction and on their ability to accumulate and store secondary metabolites that, in their turn, are important for their strategic applications, such as in aquaculture, food and pharmaceutical industries, and for power generation (Krzemińska et al., 2014; Ota et al., 2015; Santos et al., 2013).

Microalgae cultivation, mainly at commercial scale, is not yet economically viable or sustainable (Álvarez-Díaz et al., 2015; Silveira Júnior, Faustino & Cunha, 2019). Its large-scale cultivation, even in pilot projects, presents severe limitations when it comes to controlling specific environmental crop conditions, such as temperature, light, carbon dioxide concentration, pH and nutrient availability - mainly nitrogen (Bohnenberger & Crosseti, 2014). The selection of an adequate microalgae species and its optimization depends on growing–environment conditions, such as using kinetic parameters and assessing microalgal biomass at semi-industrial or industrial scale, since they can trigger a series of interdependent processes (Breuer et al., 2013).

*S. obliquus* was assessed due to its potential use for different purposes, and it explains the scientific and prospective interest in it. This interest justifies its technological assessment and the kinetic analysis applied to its growth and yield rates under different temperature, pH and NPK concentration conditions. Specific growth rate in the current analysis ranged from 0.13 ± 0.003 to 0.52 ± 0.5 day^−1^, maximum cell density was close to 101.5 ± 12.5× 10^5^ cells mL^−1^ and mean doubling time was approximately 2.78 ±1.07 days. These parameters were useful to define and analyze microorganisms’ biomass-production stages and processes. In addition, these parameters can be applied to any reactor or production system type. Yet, their usefulness is essential for the development of industrial–scale projects (Lee, Jalalizadeh & Zhang, 2015; Novoveská et al., 2016).

Li, Gao & Lin (2015) cultivated *S. obliquus* in Blue-Green medium 11 (BG11 medium) at initial inoculum of 5 × 10^4^ cell mL^−1^, and recorded specific growth rate ranging from 0.65 to 0.14 day^−1^, under light intensity of 60 and 10 µmol of photons m^−2^ s^−1^, respectively. In the present study, *S. obliquus* inoculants were grown under 28.5 µmol of photons m^−2^ s^−1^ in all experiments.

*Scenedesmus obliquus* cultivated in culture medium diluted in wastewater from olive oil production showed mean specific growth rate of 0.57 d^−1^ (Hodaifa et al., 2010). This rate is similar to the maximum rate (0.52 d^−1^) found for the inorganic medium herein cultivated at 24 °C, in alkaline pH. Similar mean rates were observed for other microalgae species, namely: *chlorella sp.* (0.57 day^−1^), *Coelastrum microporum* (0.29 day^−1^), *Rhodomonas* sp. (0.26 day^−1^), *S. pectinatus* (0.23 day^−1^) and *S. dimorphus* (0.14 day^−1^) (Bruno, Udhaya & Sandhya, 2013; Gour et al., 2016; Huerlimann, de Nys & Heimann, 2010; Mohammadi, Arabian & Khalilzadeh, 2016).

Mean maximum *S. obliquus* yield (6.21 ± 4.5 × 10^5^ cells mL^−1^ d^−1^) was similar to that observed for *S. acuminatus* crops grown in different media, including NPK (Baumgartner et al., 2013). On the other hand, the maximum yield rate of 0.99 ×10^4^ cells mL^−^1 d^−1^ was observed for *S. dimorphus* grown in BG11 urea-enriched medium (Goswami & Kalita, 2011). These different outcomes can be associated with the mean cell doubling time observed in the present study (2.78 ± 1.07 days), *i.e.,* it was shorter than that observed for other species belonging to genus *Scenedesmus*, such as *S. dimorphus* (4.91 days) *and S. quadricauda* (5.73 days)—both cultivated in BG11 medium (Gour et al., 2016). Doubling time was shorter than that observed for *Ankistrodesmus gracilis* (4.4 days) cultivated in NPK medium and in NPK+macrophytes (Sipaúba-Tavares et al., 2017).

The NPK medium confirmed the shorter doubling time, which resulted in higher growth rate and microalgae yield under the specific temperature and pH conditions used in the present study. This finding can be related to the phosphorus-nitrogen-potassium combination, which always produces higher microalgae density (Sipaúba-Tavares et al., 2017). Similar result was observed by Ammar (Ammar, 2016) for cultivar *Chlorella vulgaris* grown in NPK medium, whose doubling time was of approximately 0.52 days. Microalgae yield can increase when nitrogen and phosphorus are efficiently used for microalgal growth (Hamouda & Abou-El-Souod, 2018). Previous studies have shown that the use of NPK as single source of nutrients (nitrogen, phosphorus, and potassium) can be beneficial in comparison to the growth and biomass yield of microalgae belonging to genus *Scenedesmus* (Xin et al., 2010). The application of fertilizers, such as NPK, is a cost-effective and attractive replacement adopted to meet microalgae nutritional requirements in different cultivation processes (Nayak, Thirunavoukkarasu & Mohanty, 2016).

All growth curves in the present case fit the estimated logistic model (0.65 ≤ r^2^ ≤ 0.99, *p* < 0.05). Curves presented very short, or no lag phase, which is marked by the adaptation of cells in a pre-existing culture (N:P:K inoculum) to new environmental conditions—biomass is almost imperceptible and it can take hours or days to happen (Baumgartner et al., 2013; Lourenço, 2006). In addition, the lag phase at this growth stage, can be delayed due to the presence of non-viable cells or to physiological adjustments to the new environmental conditions (Lee, Jalalizadeh & Zhang, 2015). This finding highlight that the *S. obliquus* inoculate used in the current research presented excellent adaptation to the NPK medium and was well conditioned to the assessed environmental control variables. This very same effect was observed for *S. obliquus* cultivated in wastewater medium, which showed good adaptability to the different tested and developed cultivation conditions (Hodaifa et al., 2010).

Overall, microalgal growth presented *lag* phase (exponential) between days 2 and 7. Day 7 showed the maximum cell density recorded in the exponential growth phase. Inoculate (2.1 × 10^5^ cells mL^−1^) of *S. obliquus* cultivated in Bold’s Basal medium (BBM) medium got richer due to elements characteristic of Zn, Mn, Co and Mo. Inoculate cultivated in artificial medium (Provasolli, Schlosser and Zarrouk) showed the highest maximum growth at 10th and 15th cultivation days, respectively (Rinanti et al., 2013; Toyub et al., 2008). The inoculum of *A. gracilis* (2 × 10^5^ cel m^−1^) cultivated in NPK medium reached the maximum exponential density at the 11th cultivation day (Sipaúba-Tavares et al., 2017). *Scenedesmus acuminatus* cultivated in medium enriched with microelements presented exponential growth phase between the 3rd and 14thcultivation day (Baumgartner et al., 2013). *Scendesmus dimorphus* and *S. quadricauda* cultures took 16 days to reach the stationary phase. In addition to factors such as pH and crop temperature, the exponential phase duration mainly depends on the availability of essential nutrients (macroelements) and light (Lourenço, 2006).

*S. obliquus* growth and yield kinetics seemed to be strongly affected by both temperature and the medium dilution applied to the culture (N:P:K). Higher cell density was observed at the end of the exponential phase and at beginning of the stationary phase, within a short period-of-time (6 and 7 days) during experiments conditioned in 20 mL/L NPK, added with 1.12 g/L N, 0.32 g/L P_2_O_5_ and 0.44 g/L K_2_O. Lower NPK concentrations (20:20:20) enriched with micronutrient and vitamin B triggered the stationary phase at the 5th day of the *C. vulgaris* culture, whereas crops subjected to 0.06 and 0.08 g/L NPK started their stationary phase at the 6th and 7th cultivation days (Ammar, 2016). *Scenedesmus* sp. recorded satisfactory growth at NPK concentration of 10:26:26 added with 0.17 g/L N, 0.32 g/L P and 0.32 g/L K. This process accelerated the specific growth rate; however, it only reached the stationary phase at the 18th cultivation day (Nayak, Thirunavoukkarasu & Mohanty, 2016), *i.e.,* the exponential phase was late in comparison to that observed in the present study.

Temperature exerted strong influence on the *S. obliquus* culture grown in NPK medium. Experiments cultivated at < 24 °C showed higher mean growth (0.35 day^−1^) and yield rates (9.98 cells mL^−1^ × 10^5^ d^−1^). Hodaifa et al. (2010) found that *S. obliquus* grown in mineral medium added with 0.14 g/L N and 0.16 g/L P presented better growth rate at 29 °C, *i.e.,* 4 degrees above the maximum temperature observed for *S. obliquus* in the present study. Higher specific growth rate (1.12 day^−1^) was observed for *S. obliquus* cultivated at 30 ° C in phosphorus-enriched medium; however, the highest biomass yield was recorded at 20 °C (Martínez, Jiménez & El Yousfi, 1999). Breuer et al. (2013) observed higher initial yield and final biomass concentration at 27 °C in *S. obliquus* culture. However, this same microalgae species grown in modified BG11 medium added with 0.15 gL^−1^ NaNO_3_, 0.10 gL^−1^ K_2_HPO_4_⋅3H_2_O and 0.05 gL^−1^ Na_2_SiO_3_⋅9H_2_O showed no significant differences in growth parameters at temperature ranging from 14 °C to 20 °C and 30 °C (Xu et al., 2012). The optimum temperature-range for *S. obliquus* growth is relatively wide, because its growth rates tend to do not significantly change between thermal amplitudes ranging from 10 °C to 30 °C (Mata et al., 2013; Xin, Hong-ying & Yu-ping, 2011). A significant fraction of microalgae, including *S. obliquus*, are mesophilic organisms and they can develop at temperatures ranging from 15 °C to 40 °C (Martínez, Jiménez & El Yousfi, 1999).

A synthesis of *S. obliquus* kinetic growth and yield is described in Table 4. The aim is to better compare the kinetic parameters recorded in the present research to those available in the literature. Microalgae cultivation in NPK medium at temperature <24 °C allowed plotting a growth curve presenting higher and/or similar maximum cell density, specific growth rate and maximum yield in comparison to data in the literature. Such data are also compared to those in studies that used initial inoculums higher than that in the present study (Difusa et al., 2015; Girard et al., 2014; Goswami & Kalita, 2011; Sipaúba-Tavares et al., 2017; Xin, Hong-ying & Yu-ping, 2011). Medium pH did not have strong influence on the kinetic growth and yield of *S. obliquus* grown in inorganic fertilizer (NPK). This finding followed the wide range of pH values used in crops of different microalgae species, mainly in those of species belonging to genus *Scenedesmus*, as reported in the literature.

**Table 4:** Comparative synthesis between the growth parameters of *S. obliquus* assessed in the present study and results in studies available in the literature.

**Species**	**Cultivation conditions**			**Growth parameters**					
	**Medium**	**T** (°C)	pH	N_**0**_ (×10^4^ cel mL^−1^)	N_**max**_ (×10^5^ cel mL^−1^)	µ (day^−1^)	**T**_**2**_ (dia)	**P**_**max**_ (×10^5^ cel mL^−1^ d^−1^)	**Present research/** **References**
*Scenedesmus obliquus*	NPK medium	24.0	9.0	11.7	101.5	0.52	1.40	17.9	←**Present research**
*Scenedesmus obliquus*	NPK medium	24.0	5.0	11.7	65.5	0.42	1.60	10.7	
*Ankistrodesmus gracilis*	NPK medium	22.0		44.0	16.2	0.22	4.47		Sipaúba-Tavares et al. (2017)
*Haematococcus pluvialis*	NPK medium	23		2.6	1.4	0.17			Sipaúba-Tavares et al. (2017)
*Scenedesmus bijugatus*	Soil extract								Arumugam et al. (2013)
*Scenedesmus dimorphus*	BG11 medium	25	7.5	2,250		0.14	4.91	0.099	Goswami & Kalita (2011)
*Scenedesmus dimorphus*	BG11 medium	24		10–12		0.14	4.68		Gour et al. (2016)
*Scenedesmus obliquus*	BG11 medium	23		500		0.56			Barbera et al. (2016)
*Scenedesmus obliquus*	Vitamin B enriched medium	27.5	7.0						Breuer et al. (2012)
*Scenedesmus obliquus*	Bold’s basal	25	6.8			0.48			Chalifour & Juneau (2011)
*Scenedesmus obliquus*	Bold’s basal	22.5		100	250.0	0.26			Girard et al. (2014)
*Scenedesmus obliquus*	Zarrouk medium	25			3.1				Rinanti et al. (2013)
*Scenedesmus quadricauda*	BG11 medium	25	7.5	3,500		0.35	1.93	0.077	Goswami & Kalita (2011)
*Scenedesmus quadricauda*	BG11 medium	24		10–12		0.12	5.41		Gour et al. (2016)
*Scenedesmus* sp.	BG11 medium	25	8.0			0.36			Bakuei et al. (2015)
*Scenedesmus* sp.	BG11 medium	25	9.0	50–200	130.0	0.20	3.00		Difusa et al. (2015)
*Scenedesmus* sp.	NPK medium	25	7.0–7.5			0.24			Nayak, Thirunavoukkarasu & Mohanty (2016)
*Scenedesmus* sp.	BG11 medium	25		65		0.38			Xin et al. (2010)

**Notes.**

N_0_, Initial cell density; N_max_, Maximum cell density; μ, daily growth rate; T_2_, doubling time; P_max_, maximum yield.

Data in previous studies (Table 4) corroborated the similarity between *S. obliquus* kinetic and yield parameters in the present study. However, *S. obliquus* specific growth and yield rates recorded in the present study were higher than the averages reported in previous studies: 0.28 ±0.14 d^−1^ and 0.09 ± 0.01 ×10^5^ cells mL^−1^. d^−1^, respectively. *S. obliquus* cell doubling time (T_2_) was shorter and more effective, since it recorded mean T_2_ of 2.7 days, which was 30% shorter than the mean doubling time observed in studies available in the literature (4.07 ± 1.31 days).

## Conclusions

The kinetic study applied to microalgae belonging to species *S. obliquus* has revealed predictable and estimable growth behavior based on simple logistic models. This finding made the development of industrial-scale cultivation easier, as well as the use of these microalgae in bioprospective products. The pH ranges from 8.5 to 9.0 had stronger influence on biomass yield (Pmax). Yet, wide temperature ranges from 21 to 28 °C affected biomass yield, and lower temperatures (<24 °C) often resulted in higher mean growth and yield rates. In addition, higher NPK concentrations often led to higher biomass yield, specific growth rate and maximum cell density. This evidence becomes even more compelling if microalgal biomass is used as raw material for different biotechnological applications, mainly in aquaculture, renewable biomass energy, wastewater treatment, and in the pharmaceutical and cosmetic industries. Finally, pilot biotechnological studies conducted with *S. obliquus* deserve closer attention, since they shine light over this species’ relevant potential for valuing and bioprospecting other Amazonian species.

## Supplemental Information

10.7717/peerj.17879/supp-1 Supplemental Information 1 General data on microalgal growth

## Additional Information and Declarations

### Data Availability

The following information was supplied regarding data availability: Raw data are available as a Supplementary Files.
